# A Novel Thermo-Alkaline Stable GDSL/SGNH Esterase with Broad Substrate Specificity from a Deep-Sea *Pseudomonas* sp.

**DOI:** 10.1007/s10126-024-10308-w

**Published:** 2024-05-01

**Authors:** José Luis Rodríguez-Mejía, Itzel Anahí Hidalgo-Manzano, Luis Felipe Muriel-Millán, Nancy Rivera-Gomez, Diana X. Sahonero-Canavesi, Edmundo Castillo, Liliana Pardo-López

**Affiliations:** 1 Departamento de Microbiología Molecular, Instituto de Biotecnología, Universidad Nacional Autónoma de México, Av. Universidad 2001, Col. Chamilpa, Cuernavaca, Morelos, 62210 México; 2 Departamento de Ingeniería Celular y Biocatálisis, Instituto de Biotecnología, Universidad Nacional Autónoma de México, Av. Universidad 2001, Col. Chamilpa, Cuernavaca, Morelos, 62210 México; 3 Edificio Dr. Carlos Méndez, Centro Universitario de Investigaciones Biomédicas, Universidad de Colima, Campus Central Colima; Avenida 25 de Julio #965, Col. V. Sn. Sebastián, C.P. 28045 Colima, Colima, México; 4 IPN: CICATA Unidad Morelos del Instituto Politécnico Nacional, Blvd. de La Tecnologia 1036-P 2/2, 62790 Atlacholoaya, Morelos, México; 5 Department of Marine Microbiology and Biogeochemistry, NIOZ Royal Netherlands Institute for Sea Research, 1797AB Den Burg, P.O. Box 59, Texel, Netherlands

**Keywords:** GDSL/SGNH esterase, Autotransporter, Marine bacteria, *Pseudomonas*

## Abstract

Marine environments harbor a plethora of microorganisms that represent a valuable source of new biomolecules of biotechnological interest. In particular, enzymes from marine bacteria exhibit unique properties due to their high catalytic activity under various stressful and fluctuating conditions, such as temperature, pH, and salinity, fluctuations which are common during several industrial processes. In this study, we report a new esterase (EstGoM) from a marine *Pseudomonas* sp. isolated at a depth of 1000 m in the Gulf of Mexico. Bioinformatic analyses revealed that EstGoM is an autotransporter esterase (type Va) and belongs to the lipolytic family II, forming a new subgroup. The purified recombinant EstGoM, with a molecular mass of 67.4 kDa, showed the highest hydrolytic activity with *p*-nitrophenyl octanoate (*p*-NP C8), although it was also active against *p*-NP C4, C5, C10, and C12. The optimum pH and temperature for EstGoM were 9 and 60 °C, respectively, but it retained more than 50% of its activity over the pH range of 7–11 and temperature range of 10–75 °C. In addition, EstGoM was tolerant of up to 1 M NaCl and resistant to the presence of several metal ions, detergents, and chemical reagents, such as EDTA and β-mercaptoethanol. The enzymatic properties of EstGoM make it a potential candidate for several industrial applications.

**Supplementary Information:**

The online version contains supplementary material available at 10.1007/s10126-024-10308-w.

## Introduction

Esterases and lipases constitute a class of hydrolases (EC 3) that act on ester bonds. These lipolytic enzymes are widely distributed among eukaryotes and prokaryotes and play essential roles in biological processes, including lipid metabolism, growth, and maintenance of cellular membranes (Hausmann and Jaeger 2010). Both esterases and lipases can hydrolyze ester bonds without cofactors; however, esterases preferentially act on simple esters or short-chain triglycerides (< C6), whereas lipases hydrolyze triglycerides with long-chain fatty acids (> C10). In particular, microbial lipases and esterases are more attractive for industry than those from animals or plants because of their versatile catalytic activity, resistance to extreme conditions, easy genetic manipulation, and high-yield production (Chandra et al. 2020).

Bacterial lipolytic enzymes are grouped into 19 families according to their amino acid sequence, phylogeny, conserved motifs, and biological functions (Kovacic et al. 2019). Among these, family II includes the SGNH hydrolases, which are characterized by a distinctive four-amino-acid signature (S-G-N–H) in four conserved blocks called I, II, III, and V (Akoh et al. 2004). Additionally, a tetrapeptide (G-D-S-L) is present in the proximal amino region of the protein that includes its catalytic Ser (Kovacic et al. 2019). GDSL/SGNH esterases possess a versatile capability to cleave ester bonds and, under special conditions, exhibit synthetic or stereoselective activities. Furthermore, GDSL/SGNH esterases are considered versatile and stable under certain chemical conditions (Leščić Ašler et al. 2010; Wilhelm et al. 2011; Junmei et al. 2014; Yang et al. 2013).

An interesting characteristic of some GDSL/SGNH esterases from gram-negative bacteria is that they can be fused with the type Va secretion system. Proteins of this system are often called autotransporters (ATs) and have a typical organization consisting of a signal peptide, a passenger domain that confers the specific function of the protein, and a translocation β-domain that is embedded into the outer membrane and is fixed to the passenger domain by an α-helical linker (Henderson et al. 2004). ATs can be used for different biotechnological applications (Wilhelm et al. 2011), including the display of proteins of interest on the surface of expression hosts, as has been shown for the GDSL esterases EstA and EstP from *Pseudomonas aeruginosa* and *Pseudomonas putida*, respectively (Becker et al. 2005; Wilhelm et al. 2007a, b; Yang et al. 2010).

Marine environments are an excellent source of novel esterases (Barzkar et al. 2021). Bacteria from these ecosystems have established different levels of adaptation to the fluctuating environmental conditions, such as salinity, temperature, pressure, pH, and availability of nutrients (Muriel-Millán et al. 2021). As a part of these adaptations, esterases from marine bacteria have shown exciting potential for biotechnological applications, including in the transformation of a wide range of substrates, in the chemical and fuel industries, and in bioremediation (Debashish et al. 2005; Zhang and Kim 2010; Rao et al. 2017; Wu et al. 2013).

In this study, we report a new GDSL/SGNH esterase with an AT (EstGoM) from a marine *Pseudomonas* sp. isolated at a depth of 1000 m in the southwestern Gulf of Mexico (Rojas-Vargas et al 2023). Biochemical characterization of purified recombinant EstGoM showed activity on *p*-nitrophenyl esters (*p*-NP) with short- and medium-chain fatty acids at a wide range of temperatures (10–75 °C) and within an alkaline pH. It was stable in the presence of several inhibitors, including metal ions, chemical reagents, and detergents. The versatility of the EstGoM enzyme under extreme reaction conditions suggests its potential as a biotechnological tool in industrial cleaning and bioremediation applications.

## Materials and Methods

### Bacterial Strains and Culture Conditions

The *Pseudomonas* sp. GOM6 strain was previously isolated from a marine water sample obtained at a depth of 1000 m (GenBank accession number GCA_029537485.1), and its lipolytic activity was detected on modified lysogeny broth (LB) plates (0.5% peptone, 0.3% yeast extract, 1% tributyrin, 1% Arabic gum, and 1.3% Bacto agar) (Glogauer et al. 2011; Rojas-Vargas et al. 2023). For this study, the GOM6 strain was routinely cultured in standard LB medium (1% tryptone, 0.5% yeast extract, and 1% NaCl) with incubation at 30 °C. The *Escherichia coli* DH5α (GIBCO-BRL) and BL21(DE3) (Invitrogen) strains were grown in LB medium and incubated at either 30 or 37 °C, depending on the experiment.

### Construction of a *Pseudomonas* sp. GOM6 Genomic Library and Identification of Lipolytic Genes

Genomic DNA of *Pseudomonas* sp. GOM6 was extracted using the Quick-gDNA MiniPrep Kit D30245 from Zymo Research (Irvine, CA, USA) following the protocol specifications. The genomic DNA was partially digested using the Bsp143l isoschizomer of *Sau3A*I (Thermo Fisher Scientific™; Waltham, MA, USA). The digested genomic DNA was separated by electrophoresis in a 0.8% agarose gel, and only fragments between 3 and 6 kb were excised from the gel and recovered using the GeneJET Gel Extraction Kit from Thermo Fisher Scientific™_._ The DNA fragments were ligated into the pUC19 plasmid at a 1:10 molar ratio of vector/insert using T4 DNA ligase (Thermo Scientific™) and following the recommended protocol for sticky-end ligation. The ligation reaction mixture was transformed into electrocompetent *E. coli* DH5α cells, which were then plated on modified LB plates supplemented with 1% tributyrin as a lipidic substrate (Glogauer et al. 2011), 0.5 mM isopropyl-β-D-thiogalactopyranoside (IPTG), and 100 µg∙mL^−1^ ampicillin. The plates were incubated at 30 °C for 48 h and 4 °C for 3 days. Colonies surrounded by clear halos were considered potential clones with lipolytic activity. Plasmids from positive clones were purified using the GeneJET Plasmid Miniprep Kit from Thermo Scientific (Waltham, MA, USA). The purified plasmids were sequenced at the DNA Synthesis and Sequencing Unit IBt-UNAM using the pUC/M13 reverse primer. The obtained sequences were annotated using the RAST server (Aziz et al. 2008).

### Computational Analysis of the Lipolytic EstGoM Protein

Sequences homologous to *Pseudomonas* sp. GOM6 EstGoM were identified using BLASTP (https://blast.ncbi.nlm.nih.gov/) within the nonredundant (nr) database. For the identification of domains in the EstGoM protein, the SUPERFAMILY server (http://supfam.org/) was used (Pandurangan et al. 2018).

Classification of EstGoM within the lipase/esterase families was performed by constructing a phylogenetic tree with the amino acid sequences of close homologs identified in the nr database, the sequences of proteins cited by Kovacic et al. (2019), and the sequences of some experimentally characterized SGNH esterases with and without natural ATs (Talker-Huiber et al. 2003; Yang et al. 2013; Wicka et al. 2016; Shakiba et al. 2016; Petrovskaya et al. 2015; Cai et al. 2017). The MEGA X program was used for multiple sequence alignment with the default parameters for the Muscle tool (Kumar et al. 2018). The phylogenetic analysis to determine membership in the lipase family was conducted using the UPGMA method with a bootstrap test (3000 replicates), and the evolutionary distances were computed using the Poisson correction method. The Jalview program ver 2.11.1.3 was used to visualize the sequence alignments.

### Computational Modeling of EstGoM

The tertiary structure of EstGoM was predicted using SWISS-MODEL (https://swissmodel.expasy.org/) with automated mode parameters (Waterhouse et al. 2018), and the structure of *P. aeruginosa* EstA was used as a template (PDB: 3KVN) (van den Berg 2010). An EstGoM.pdb archive was obtained for complete and split model emulation. The suite software Swiss-PdbViewer version 4.1.0 was used to analyze the three-dimensional structure.

### EstGoM Overexpression and Purification

The EstGoM-coding gene was amplified by PCR using the oligonucleotides Fest3Nde (5′-ATCATTACATATGAAGCGAGTTTTGACG-3′) and RestEcoR (5′-AAATTGAATTCT TATTACCAGTCGAGCACCAG3′). The PCR product was digested with NdeI and EcoRI, gel purified, and inserted into the pET-28a ( +) vector (Novagen) previously digested with the same enzymes. The resulting plasmid (pET-EstGoM) was verified by DNA sequencing and used to transform the *E. coli* BL21 (DE3) strain.

The *E. coli* BL21(DE3) strain carrying the pET-EstGoM plasmid was grown in 1 L of LB medium containing 30 µg∙mL^−1^ kanamycin with incubation at 37 °C and 200 rpm. When an optical density (OD) of approximately 0.5 was reached, IPTG was added to the culture at a final concentration of 0.5 mM, and the cells were further incubated at 37 °C and 200 rpm for 3 h. The culture was divided into 100 mL aliquots, and the cells were harvested by centrifugation for 10 min at 5000 rpm at 4 °C. The cell pellets were stored at -20 °C until further use. Three pellet samples were used for protein purification; each one was resuspended in 6 mL of lysis buffer (pH 8.0; 50 mM NaH_2_PO_4_, 300 mM NaCl, 10 mM imidazole, 2% sarkosyl, 10% glycerol, 1 mM β-mercaptoethanol, 1 × cOmplete™ Protease Inhibitor Cocktail -Roche), and lysozyme was added at a final concentration of 1 mg∙mL^−1^. The samples were incubated on ice for 1 h, and then, the cells were disrupted by ultrasonication in a Virtis Virsonic 60 Ultrasonic Cell Disruptor with six 10 s cycles of ultrasonication at 8 watts. The crude lysates were centrifuged at 13,000 rpm for 30 min at 4 °C, and the supernatants were recovered. The EstGoM protein was purified by affinity chromatography using nickel resin (Ni–NTA Agarose, Qiagen) and eluted with a lysis buffer containing 250 mM imidazole. Dialysis was performed using a 3 kDa MW Amicon Ultra-0.5 (Merck, Germany) with a concentrated buffer containing 50 mM NaH_2_PO_4_, 300 mM NaCl, and 0.05% Triton X-100, with the pH adjusted at 8.0, as described by Wicka et al. (2016). The purified EstGoM was visualized by sodium dodecyl sulfate polyacrylamide gel electrophoresis (SDS‒PAGE) (Fig. S1A). The purity of EstGoM was estimated by densitometry analysis of SDS-PAGE gels using the ImageJ-NIH software (Schneider et al. 2012).

### Detection of 6xHis-EstGoM by Western Blotting

Western blot analysis was performed by loading aliquots of purified 6xHis-EstGoM and C12DO (positive control) (Rodriguez-Salazar et al. 2020) proteins onto a 10% polyacrylamide gel. Proteins were transferred to a nitrocellulose membrane and blocked with 5% skim milk in Tris-buffered saline (TBS; pH 7.5) for 1 h at room temperature. The membrane was washed with TBS-0.1% Tween 20 (TBST) and probed with an alkaline phosphatase-conjugated anti-6xHis tag antibody (Abcam, UK) at a 1:5000 dilution overnight at 4 °C. Then, the membrane was washed with TBST and developed with SuperSignal West Pico Chemiluminescent Substrate (Thermo Fisher Scientific™).

### Enzyme activity and substrate specificity of EstGoM

The hydrolytic activity of purified EstGoM was assayed using the following *p*-NP esters as substrates: *p*-nitrophenyl butyrate (C4:0), *p*-NP valerate (C5:0), *p*-NP caprylate (C8:0), *p*-NP caprate (C10:0), *p*-NP laurate (C12:0), *p*-NP palmitate (C16:0), and *p*-NP stearate (C18:0) (Sigma, Mo., USA). Stock solutions of *p*-NP butyrate, valerate, and caprylate were prepared in isopropanol. The *p*-NP caprate, laurate, palmitate, and stearate esters were dissolved in a 4:1 isopropanol to acetonitrile mixture, as previously described (Glogauer et al. 2011). The ability of EstGoM to liberate *p*-nitrophenol from acyl esters was measured spectrophotometrically at 400 nm in a BioTek Synergy H1 96-well microplate reader (Corning®, USA). The total volume of the reaction was 0.2 mL, consisting of 50 mM Tris–HCl buffer (pH 8.0), 6.25 × 10^–4^ mg⋅mL^−1^ protein, and 0.5 mM substrate (*p*-NP esters). The reactions were followed for 4 min at 30 °C. A control background of auto-hydrolyzed substrate was considered for all reactions assayed. Esterase activity was reported in enzyme activity units; one unit of enzyme activity (U) was defined as the amount of enzyme that released 1 μmol of *p*-nitrophenol min^−1^ at 30 °C.

### Optimum pH, Temperature, and NaCl Concentration for EstGoM Esterase Activity

The optimum pH for EstGoM esterase activity was determined for the hydrolysis of *p*-NP caprylate using Britton-Robinson buffer over a pH range of 7 to 11 as described by Wicka et al. (2016). The optimum temperature for EstGoM esterase activity was determined by assaying at temperatures ranging from 10 to 75 °C (pH 8.0); the kinetic assays were monitored at different temperatures for 4 min in a Beckman DU 650 Spectrophotometer equipped with a Peltier system for temperature control. To evaluate the effect of NaCl on enzyme activity, the assays were performed at NaCl concentrations of 0.3, 0.5, 1, 1.5, 2, 3, and 4 M. Reactions without NaCl were included as controls.

### Effect of Metal Ions, Detergents, and Denaturing/Chelating Agents on the Catalytic Activity of EstGoM

The effect of different concentrations of metal ions and detergents on the EstGoM enzymatic activity was determined in the standard reaction buffer as described in the enzyme activity section, using *p*-NP caprylate as the substrate. The assays were performed at concentrations of 5 mM for Na^1+^, K^1+^, Mg^2+^, Ba^2+^, Mn^2+^, Cu^2+^, Ni^2+^, Sr^2+^, Hg^2+^, Ca^2+^, Co^2+^, and Zn^2+^ (Cieśliński et al. 2007; Shakiba et al. 2016). Similarly, the effect of detergents on EstGoM esterase activity was assayed. All reactions were carried out in the presence of 0.5% (w/v) of each of the following detergents: 3-((3-cholamidopropyl)dimethylammonium)-1-propanesulfonate (CHAPS), hexadecyltrimethylammonium bromide (CTAB), sarkosyl, SDS, sodium deoxycholate, Triton X-100, Tween-20, and Tween-80. All reactions were independently monitored and performed in triplicate. Reactions without metal ions or detergents were included as control tests**.**

### EstGoM Stability to Temperature, pH, and Detergents

The stability of EstGoM was determined by measuring its residual enzymatic activity after a specific incubation time at different pH values, temperatures, or detergent concentrations. These assays were carried out by incubating 6.25 × 10^–4^ mg⋅mL^−1^ purified EstGoM for 30 and 60 min in the presence of each selected condition. For the EstGoM pH stability assay, pH values in the range of 5–12 were selected. For testing EstGoM stability in the presence of detergents, CHAPS, CTAB, sarkosyl, SDS, sodium deoxycholate, Triton X-100, Tween-20, and Tween-80 were used at a concentration of 0.5% (w/v). The stability of the purified protein at different temperatures was assayed from 10 °C to 55 °C at 5 °C intervals. After incubation, standard enzyme activity assays were carried out as described above. The different enzyme activities were considered residual enzymatic activity for calculations. All assays were carried out in triplicate, and controls were established at optimum pH and temperature and in the absence of detergents during the incubation period.

## Results

### EstgoM Gene Identification

Recently, we reported the genome of *Pseudomonas* sp. GOM6, a lipolytic strain isolated from deep-sea water (Rojas-Vargas et al 2023). To identify genes potentially involved in the lipolytic activity observed in the GOM6 strain, we constructed a genomic library (see Materials and Methods). Approximately 5346 colony-forming units (CFUs) were screened on modified LB plates supplemented with tributyrin. After 2 days of incubation at 30 °C, two *E. coli* DH5α clones exhibited clear halos indicative of hydrolytic activity against tributyrin. In addition, to select clones with lipolytic activity at cold temperatures, the plates were further incubated at 4 °C; after 3 days, one colony also showed a hydrolytic halo (clone three; Fig. S2). The genomic DNA region inserted in the plasmid of this clone was 4.5 kb in size. Functional genome annotation by RAST identified five genes in the GOM6 DNA insert (Fig. S3), one of which encoded a phospholipase/lecithinase/hemolysin protein of 633 amino acid residues with a molecular mass calculated to be 67.4 kDa (here named EstGoM), which could be associated with the activity of *E. coli* clone three on tributyrin (Fig. S2).

### Bioinformatic analysis of EstGoM

A BLASTP search revealed that the closest homolog of EstGoM is an AT domain-containing SGNH/GDSL hydrolase family protein from *Pseudomonas* sp. BMS12 (WP_068824744.1) (Mishra et al. 2016), with an identity of 84% (100% coverage and an *E*-value of 0.0). Interestingly, neither this protein nor other close homologs of EstGoM with lower identity have been functionally characterized, except for EstA from *P. aeruginosa* (Wilhelm et al. 1999), which has an identity of 50% with EstGoM (100% coverage, *E*-value of 0.0) (Table S1).

**Fig. 1:**
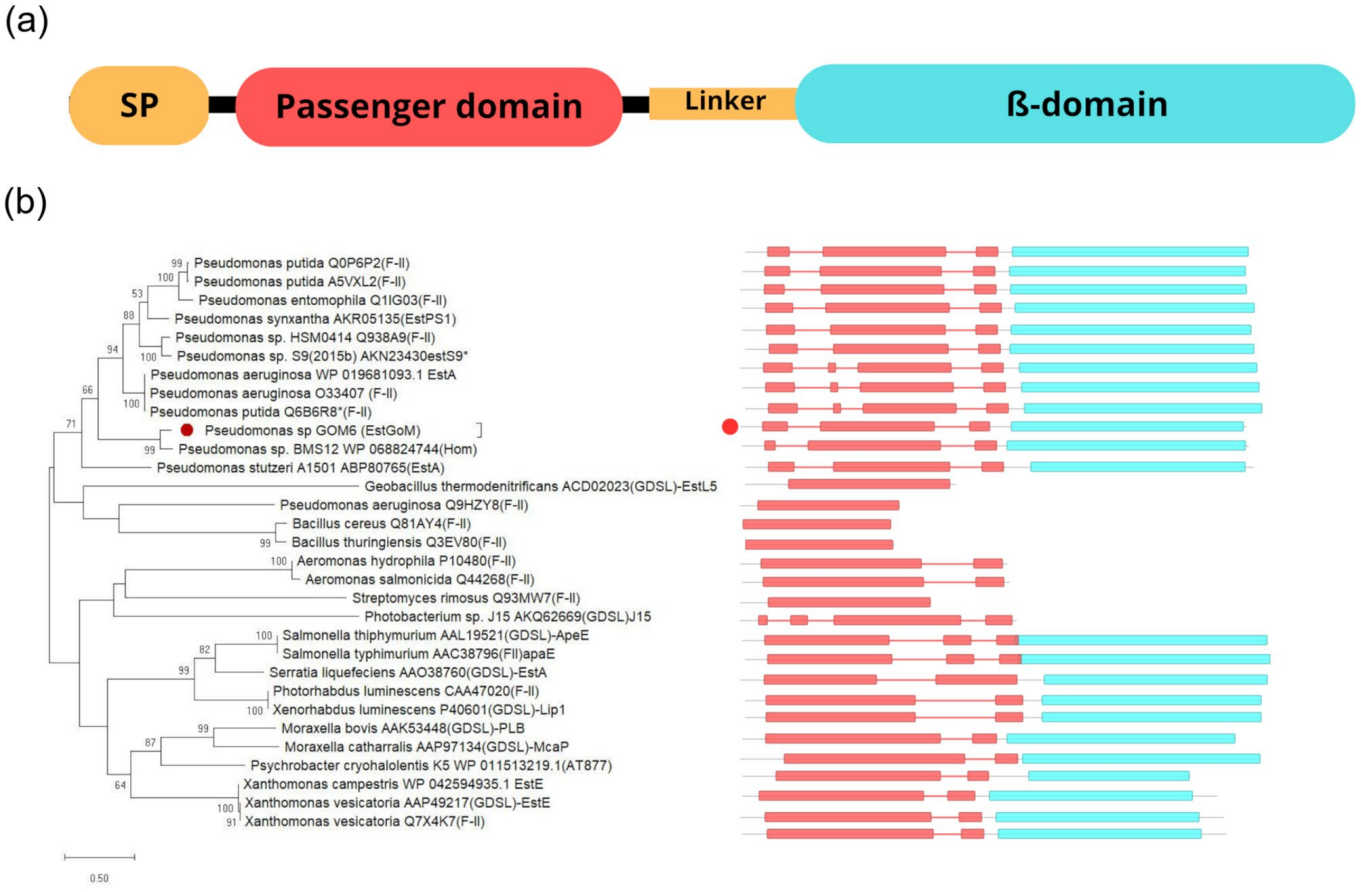
EstGoM is an autotransporter esterase belonging to family II of lipolytic enzymes. **a** Schematic representation of the type Va secretion system, which is composed of a signal peptide (SP), a passenger domain with specific activity, a linker, and a β-domain that forms a β-barrel that anchors the protein to the bacterial outer membrane. **b** Phylogenetic tree of EstGoM and homologs belonging to family II of lipolytic enzymes. The analysis was performed using the maximum likelihood tree test in MEGA X software and the amino acid sequences of EstGoM and its close homologs. The red circle shows the position in the phylogenetic tree and the secondary structure of EstGoM

The analysis of the EstGoM amino acid sequence on the SUPERFAMILY server revealed that this protein has two domains: an SGNH hydrolase superfamily domain (SCOP IDE: 3,001,315) at the N-terminus covering residues 29 to 313 (E-value 1.39E-14) and an AT superfamily domain (SCOP ID: 4,001,769) between residues 339 and 629 (E-value of 1.24e-40). Alignment of the amino acid sequence of the SGNH hydrolase domain of EstGoM with those of its relatives revealed the presence of the tetrapeptide GDSL at the proximal amino region instead of GXSXG conserved in the true lipases and the amino acids of the catalytic triad (Ser34, His302, and Asp299) (Fig. S4). The organization of EstGoM domains resembles that of proteins secreted from gram-negative bacteria classified into the type Va secretion system (Fig. 1a). The presence of the SGNH domain and the GDSL motif in EstGoM suggested that this protein belongs to family II of lipolytic enzymes known as GDSL/SGNH. This enzyme presents four conserved blocks typical of the SGNH hydrolase family (I, II, III, V), and only this block included a strictly conserved amino acid essential for the catalysis (Fig. S4) (Akoh et al. 2004; Kovacic et al. 2019; Hausmann and Jaeger 2010). To confirm this, we constructed a phylogenetic tree with 208 proteins covering all 19 presently described families. We also included the amino acid sequences of lipolytic proteins similar to EstGoM for which experimental characterization has been reported (Talker-Huiber et al. 2003; Nicolay et al. 2012; Petrovskaya et al. 2015; Yang et al. 2013; Shakiba et al. 2016; Cai et al. 2017). This analysis confirmed that the EstGoM protein belongs to family II of lipases/esterases (Fig. S5).

To obtain deep insight into the relationships of EstGoM with nearby proteins, we performed a second analysis with only members of the branch where EstGoM occurs, most of which belong to family II. As shown in Fig. 1b, these proteins were divided into two groups, both including members with and without an AT. As expected, EstGoM clustered with other *Pseudomonas* proteins; however, it and its closest relative from *Pseudomonas* sp. BMS12 were in a different branch, indicating the formation of a probable new subgroup (Fig. 1b). These results strongly suggest that EstGoM is a new esterase belonging to family II of lipolytic enzymes.

### Homology Modeling of the EstGoM Protein

**Fig. 2:**
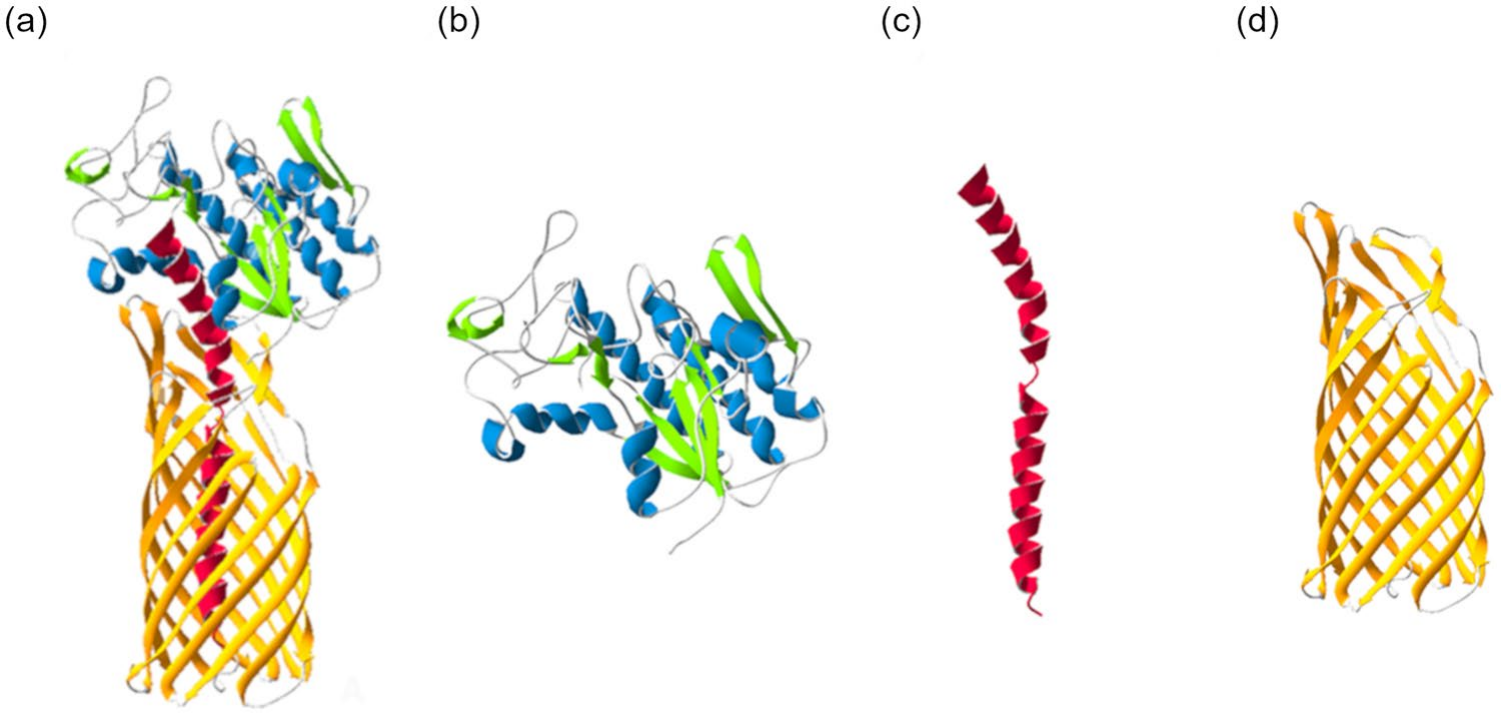
Modeling of the three-dimensional structure of EstGoM. **a** Predicted structure of the full-length EstGoM. The β-barrel (yellow) and passenger (blue and green) domains are connected by a linker (red) that resides in the lumen of the translocation domain. **b** Structure of the EstGoM esterase domain that presents a common α/β SGNH hydrolase fold, which consists of central β-sheets surrounded by α-helices. **c** The EstGoM linker is modeled as a long α-helix. **d** Structure of the β-barrel transporter of EstGoM that is composed of 12 antiparallel β-sheets. The EstGoM model was created from the EstA structure of *P. aeruginosa* (PDB: 3KVN) using SWISS-MODEL (https://swissmodel.expasy.org/)

To determine the possible conformation of EstGoM, we predicted its tertiary structure using the SWISS-MODEL webserver (https://swissmodel.expasy.org/) with automated mode parameters, where the monomeric EstA of *P. aeruginosa* (PDB: 3KVN) was the principal template (van den Berg 2010) with 51.40% identity and 0.96 coverage. The QMEANDisco Global score of the 3D model of full length EstGoM was 0.78 ± 0.5 (range 0–1) and exhibited a typical conformation of type Va ATs (Fig. 2a), in which the passenger domain (esterase) is secreted outside the cell through the AT domain but remains connected to it by the linker within the barrel lumen (Meuskens et al. 2019). The esterase domain exhibited a globular fold in which α-helices surrounded four parallel β-sheets. (Fig. 2b). The esterase is linked to the β-barrel by a region of amino acids (linker) that form a long alpha helix (Fig. 2c), and the AT is composed of 12 antiparallel β-sheets creating a narrow channel (Fig. 2d). Collectively, these results indicate that EstGoM and EstA have similar structures, with a QMEANDisco Global score of 0.78 ± 0.5.

A 3D image analysis using DeepView/Swiss-PdbViewer was used to show the particular characteristics of the catalytic core of SGNH esterases and their conservation in EstGoM. A close view of the catalytic site of EstGoM shows that the catalytic triad is formed by the amino acids Ser34, His302, and Asp299, a distinctive core for SGNH esterases, with a perpendicular orientation with two extra proton donors, G99 and N157 (Fig. S6) (Mølgaard et al. 2000).

### Expression and Purification of the EstGoM Protein

The expression and purification conditions described in the “Materials and Methods” section allowed us to obtain a soluble 6xHis-tagged EstGoM protein with an estimated molecular mass of 67 kDa, which was visualized by SDS‒PAGE and detected by Western blotting using an anti-His antibody (Fig. S1b). The average concentration of the purified protein was 0.5 mg⋅mL^−1^. Densitometry of SDS‒PAGE images revealed that the EstGoM obtained was 79% pure (Fig. S1a). Moreover, evaluation of the purification process determined a purification factor of 24, global yield of 15%, and specific activity of protein preparations of 4320 U⋅mg protein^−1^ (Table S2).

### Biochemical Characterization of the EstGoM Esterase

#### Substrate Specificity

**Fig. 3:**
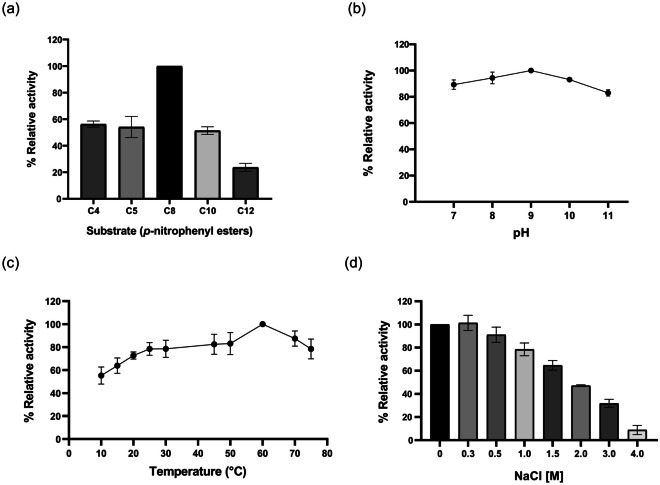
Biochemical characterization of purified EstGoM. **a** Determination of EstGoM substrate specificity. The relative activity was calculated assuming the highest activity observed with *p*-nitrophenyl caprylate (C8) as 100%. **b** Determination of the optimum pH for EstGoM activity with* p*-NP C8. **c** Optimum temperature for EstGoM activity with *p*-NP C8. **d** EstGoM activity in the presence of NaCl. In all panels, the data are the mean values of three independent experiments performed in triplicate. Error bars, standard deviation (SD)

The evaluation of the ability of EstGoM to hydrolyze *p*-NP esters bearing different fatty acids at 30 and 60 °C showed that this enzyme prefers medium-chain esters. The maximum hydrolysis rate observed at 60 °C was for *p*-NP caprylate (C8:0; 4320 U⋅mg protein^−1^), and lower transformation rates were observed for *p*-NP butyrate (C4:0), *p*-NP valerate (C5:0), *p*-NP caprate (C10:0), and *p*-NP laurate (C12:0) (2400, 2240, 2240, and 960 U⋅mg protein^−1^, respectively). For long-chain *p*-NP esters (*p*-NP palmitate (C16:0) and stearate (C18:0)), no hydrolytic activity was detected. The relative activity profile is presented in Fig. 3a.

#### Determination of Optimal pH, Temperature, and NaCl Concentration

The enzymatic activity profiles of EstGoM as a function of pH, temperature, and NaCl concentration were determined for the hydrolysis of *p*-NP caprylate. We failed to measure the EstGoM activity at acidic pH values due to the spontaneous hydrolysis of *p*-NP substrates under these conditions; therefore, we only evaluated the enzymatic activity in the pH range from 7 to 11. The maximum EstGoM activity (100%) was at pH 9. The enzyme retained more than 80% of its activity at pH 7 and 11 and showed minimal activity loss at pH 8 and 10 (95% and 90%, respectively) (Fig. 3b).

With regard to the effect of temperature on the catalytic activity of EstGoM, the enzyme was active over the whole analyzed range of temperatures (10–75 °C), showing better catalytic activities in the high-temperature range, with optimum activity at 60 °C and relative activities above 80% in the range of 25–75 °C. Notably, EstGoM retained approximately 50–70% of its catalytic activity at low temperatures between 10 and 20 °C (Fig. 3c).

The effect of different concentrations of NaCl on the catalytic activity of EstGoM was determined in a range of 0.3–4 M NaCl and compared to the activity under control conditions in the absence of NaCl (Fig. 3d). The assay results showed the halotolerant properties of EstGoM, as the enzyme retained at least 80% of its catalytic activity under NaCl concentrations up to 1 M. Moreover, although a constant decrease in catalytic activity was observed at concentrations higher than 1 M, EstGoM retained at least 10% of its activity at extremely high concentrations of NaCl (4 M) (Fig. 3d).

#### Effect of Metal Ions, Detergents, and Denaturing or Chelating Agents on the Catalytic Activity of EstGoM

**Fig. 4:**
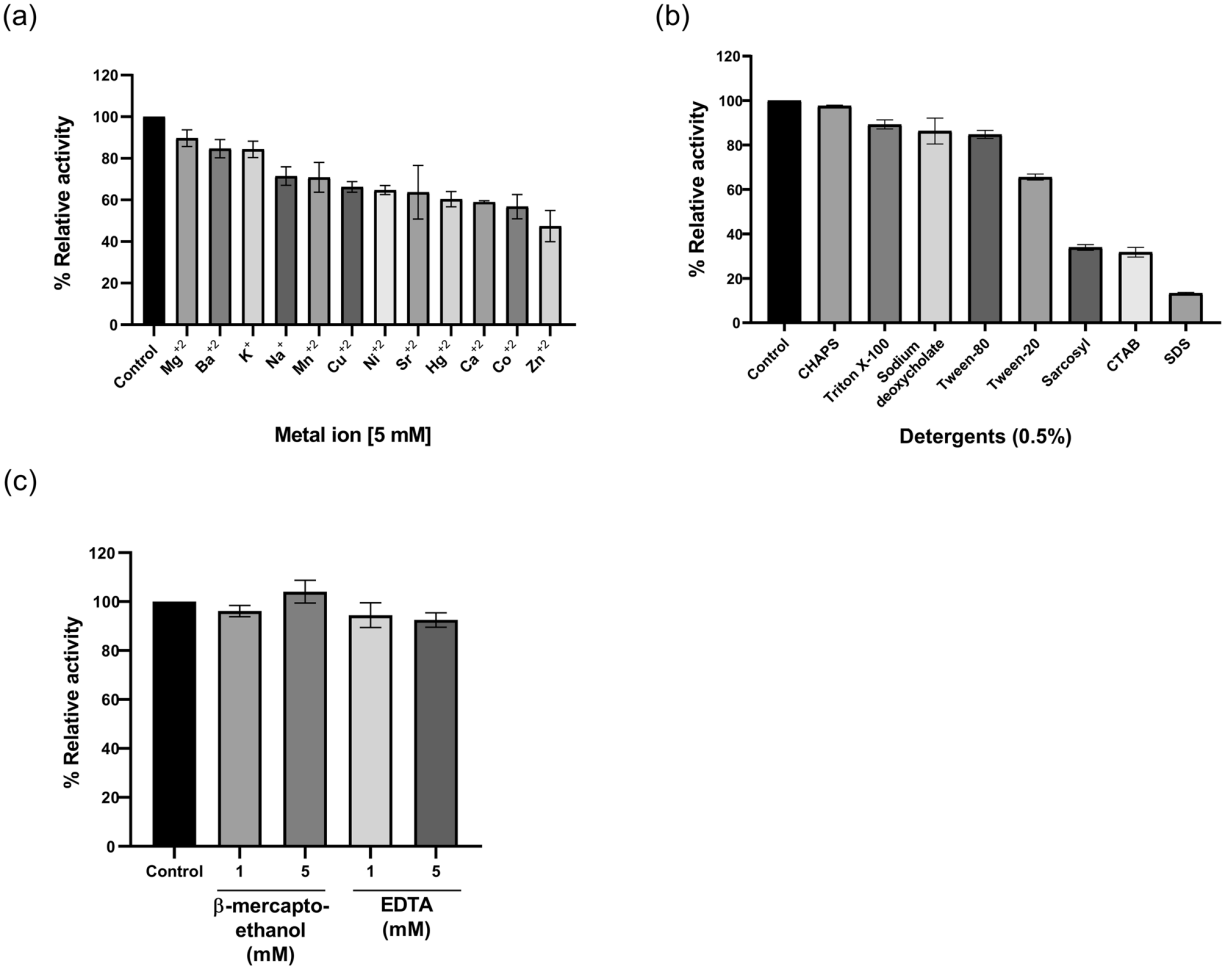
Effect of metal ions, detergents and chemical reagents on EstGoM activity. **a** Relative activity of EstGoM in the presence of several cationic metal ions (5 mM). **b** EstGoM activity in the presence of detergents (0.5%). **c** Activity of EstGoM in the presence of EDTA and β-mercaptoethanol at 1 and 5 mM. For all panels, the activity of EstGoM was determined with *p*-NP C8. The data are the mean values of three independent experiments performed in triplicate. Error bars (SD)

The effect of metal ions on EstGoM activity was evaluated for the hydrolysis of *p*-NP caprylate. The results showed moderate tolerance to different mono- or divalent metal ions at 5 mM. Indeed, the enzyme retained 80% of its activity in the presence of Mg^2+^, K^+^, and Ba^2+^, while only 60% of its activity was retained in the presence of Na^+^, Mn^2+^, Cu^2+^, Ni^2+^ Sr^2+^, Hg^2+^, and Ca^2+^. Furthermore, Zn^2+^ and Co^2+^ decreased the activity to 50 and 55%, respectively (Fig. 4a).

The relative activity of EstGoM was evaluated in the presence of detergents at a concentration of 0.5% (w/v) (Fig. 4b). All the detergents decreased EstGoM activity; however, while the relative activity with Triton X-100, sodium deoxycholate or Tween 80 remained above 80%; the relative activity with Tween 20 was only 60%. The relative activity of EstGoM dropped dramatically with amphiphilic detergents such as sarkosyl (34%) and CTAB (30%) and in the presence of SDS (10%) (Fig. 4b).

The effect of two concentrations (1 and 5 mM) of β-mercaptoethanol and EDTA on the activity of EstGoM was also evaluated. These compounds are denaturing and chelating agents, respectively, and they are routinely used in the extraction and purification of enzymes. Interestingly, EstGoM retained more than 90% of its relative activity in the presence of both β-mercaptoethanol and EDTA at concentrations of 1 and 5 mM (Fig. 4c).

#### Effect of Temperature, pH, and Detergent on EstGoM Stability

**Fig. 5:**
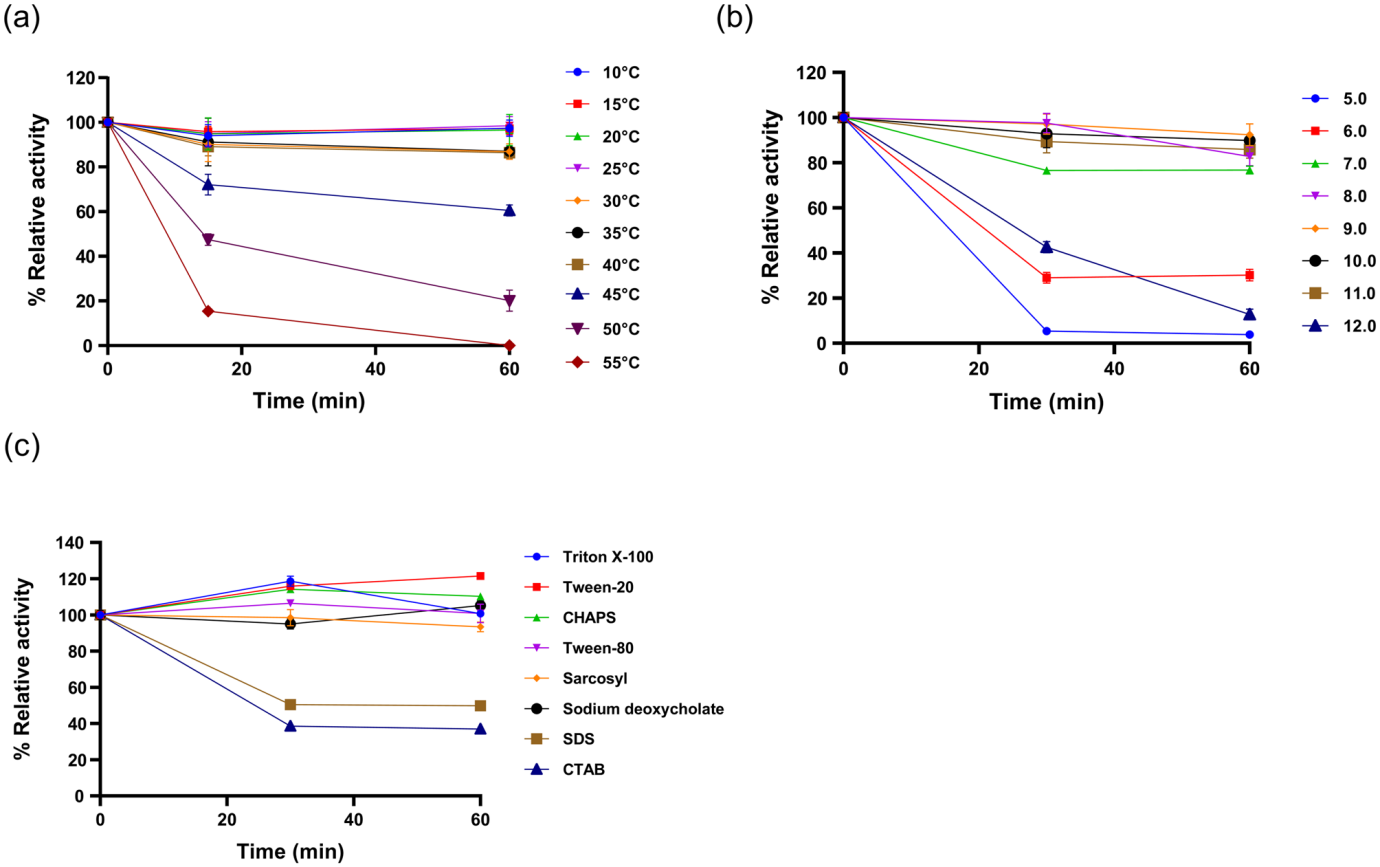
Effects of pH, temperature, and detergents on EstGoM stability. **a** Thermal stability assays were performed in a range of 10–55 °C. **b** pH stability of EstGoM at pH values from 5 to 12. **c** EstGoM stability after incubation with different detergents (0.5%). For all panels, the activity of EstGoM was determined with *p*-NP C8. The data are the mean values of three independent experiments performed in triplicate. Error bars (SD)

The stability assays showed that EstGoM is a highly stable enzyme at temperatures between 10 and 40 °C. Indeed, after 1 h of incubation at temperatures between 10 and 25 °C, EstGoM was entirely stable (100% relative activity), and at temperatures of 25–40 °C, EstGoM retained 90% of its relative activity. At 45 °C, the relative activity was 60%, while at 50 °C, the relative activity fell to 20%, with total loss of activity observed at 55 °C after 1 h of incubation (Fig. 5a). Similarly, the stability of EstGoM was evaluated in the pH range of 5–12 for 1 h. Importantly, EstGoM was highly stable in the pH range of 7–11, retaining a relative activity between 80 and 100%. Conversely, the stability at pH 5, 6, and 12 was drastically reduced, with 95, 80, and 90% activity loss (Fig. 5b).

Finally, the stability of EstGoM was evaluated under storage with detergents (0.5% w/v) for 1 h. Surprisingly, EstGoM exhibited complete stability in the presence of Tween 80, sarkosyl, and sodium deoxycholate and even improved stability in the presence of Triton X-100, CHAPS, and Tween 20 (120% relative activity). In contrast, SDS and CTAB reduced the activity to 45 and 40%, respectively (Fig. 5c).

## Discussion

In the last decade, marine microorganisms and their proteins have demonstrated potential for many biotechnological applications. Most esterase proteins were identified and investigated at least forty years ago (Trincone 2010). An increase in the demand for new biocatalytic tools has led to new explorations in marine niches, where microorganisms are considered a source of proteins with new adaptations (Fang et al. 2014; Sana 2015). Here, by functional screening of a genomic library of the marine *Pseudomonas* sp. GOM6 strain, we identified a novel esterase with an AT that was active under several physicochemical conditions.

Phylogenetic analysis revealed that EstGoM is a GDSL/SGNH hydrolase belonging to the II lipolytic enzyme family (Fig. 1b), and its closest homolog is an uncharacterized esterase from *Pseudomonas* sp. BMS12, a strain isolated from lake rhizosphere sediments in India (Mishra et al. 2016). The separation of EstGoM from its homologs in a different branch suggests the formation of a new subgroup for this type of protein (Fig. 1b). However, more detailed analyses of EstGoM and its homologs are needed to understand the evolutionary relationship among these esterases.

The organization of domains in EstGoM indicates that it is a type Va AT protein. Type Va ATs constitute a simple secretion mechanism in gram-negative bacteria, consisting of a single polypeptide containing a β-barrel domain that resides in the outer membrane and translocates the passenger domain outside the cell (Meuskens et al. 2019). The β-barrel and passenger domains are connected by an α-linker, which in most ATs contains an intein-like motif allowing the cleavage and release of the passenger domain into the surrounding environment. However, lipolytic passenger domains remain attached to the translocator domain, performing physiological functions (van Ulsen et al. 2018). For instance, in *P. aeruginosa*, EstA localizes on the cell surface and is required for the synthesis of rhamnolipid surfactants and for biofilm formation (Wilhelm et al. 2007a, b). Given that EstGoM is structurally similar to *P. aeruginosa* EstA (Fig. 2a), it is tempting to speculate that in *Pseudomonas* sp. GOM6, EstGoM could remain attached to its β-barrel on the cellular surface and play biological roles related to growth promotion (e.g., niche colonization and carbon source supply) or metabolism (Hausmann and Jaeger 2010).

Some type-Va ATs have been used to display recombinant proteins on the cell surface or in outer membrane vesicles for vaccine development, biocatalysis, and biosensor design (Jong et al. 2014; Jose et al. 2009; Tozakidis et al. 2020; Daleke-Schermerhorn et al. 2014). In addition, ATs may positively affect the activity of recombinant proteins. For instance, a variant of the *P. synxantha* EstPS1 esterase lacking an AT exhibited a reduction in thermostability and catalytic activity compared with full-length EstPS1 (Cai et al. 2017). Thus, the AT domain of EstGoM could favor its enzymatic activity under the conditions tested here and could be explored to produce other proteins by changing the passenger domain.

A search for EstGoM homologous proteins with experimental data yielded only a few characterized GDSL esterases with ATs from 1999 to 2017, mainly from *Pseudomonas* species (Table 1). However, unlike most reported carboxylesterases, EstGoM displayed maximum activity not against short-chain esters but rather against medium-chain esters. For instance, the best substrate for EstA from *P. aeruginosa* PAO1 was *p*-NP C6, and for *P. stutzeri* A15 EstA, *P. synxantha* PS1 EstPS1, and *Pseudomonas* sp. S9 EstS9N, it was *p*-NP C4 (Table 1), whereas EstGoM showed its highest activity toward C6. In addition, EstGoM was capable of hydrolyzing *p*-NP C4, C5, C10, and C12 (Fig. 3a). This unique broad specificity makes this protein stand out among different carboxylesterases belonging to the same family (Table 1). The broad specificity for acyl chain length and other interesting functional properties expands the potential of EstGoM for biotechnological applications.

**Table 1:** Comparison of the enzymatic activity of EstGoM and other esterases

Reference	Protein/strain	Substrate specificity and activity reported	Substrate specificity and activity of EstGoM
Wilhelm et al. 1999	EstA/*Pseudomonas aeruginosa* PAO1	100% relative activity against *p*-NP caproate (C6), 30 °C, cellular fractions	100% relative activity over p-NP caprylate (C8), 60 °C, purified
Talker-Huiber et al. 2003	*Xv*_EstE/*Xanthomonas vesicatoria* DSM 50861	28.7 units mL^−1^, 27 units mL^−1^, 22.6 units mL^−1^ over C3, C6 and C4, respectively; 30 °C, purified	1.5 units mL^−1^, 1.4 units mL^−1^, 2.7 units mL^−1^, 1.4 units mL^−1^, 0.6 units mL^−1^ over C4, C5, C8 C10 and C12, respectively; 60 °C, purified
Nicolay et al. 2012	EstA/*Pseudomonas stutzeri* A15	100% relative activity against *p*-NP butyrate (C4), 37 °C, purified	100% relative activity against *p*-NP caprylate (C8), 60 °C, purified
Petrovskaya et al. 2015	EstPc (fused with AT877 autotransporter)/*Psychrobacter cryohalolentis* K5T	Maximum relative activity (100%) against *p*-NP decanoate (C10) at 50 °C and pH 9, also active in pH 7.2–9.5, total membrane fraction	Maximum relative activity (100%) against *p*-NP caprylate (C8) at 60 °C and pH 9.0, also active on pH 7.0–11.0, purified
Wicka et al. 2016	EstS9N/*Pseudomonas* sp. S9	100% relative activity against *p*-NP butyrate (C4), 25 °C; km 0.0095 over p-NP C4 at 25 °C, active on pH 7.5–9.5, purified	100% relative activity against *p*-NP caprylate (C8), 60 °C, active on pH 7.0–11.0, purified
Cai et al. 2017	EstPS1/*Pseudomonas synxantha* PS1	100% relative activity against* p*-NP butyrate (C4), also against C2, C6 and C8 but lower than that over C4, pH 8 at 60 °C. Additionally, hydrolysis of pyrethroids: carbaryl > fenpropathrin > trans-cypermethrin > cis-cypermethrin > fenvalerate > bifenthrin. km μM 43 over C4 p-NP butyrate, purified	100% relative activity against *p*-NP caprylate (C8), pH 9.0 at 60 °C, purified

Indeed, among industrial applications, the ability of EstGoM to retain high enzymatic activity in a pH range of 7–11 (80%) and show relevant activity in a temperature range of 10–75 °C (T_opt_ = 60 °C) facilitates its use in the detergent industry (St. Laurent et al. 2007).

In addition, some other interesting characteristics make EstGoM a unique enzyme within the carboxylesterase family to which it belongs, making it a potential candidate for industrial applications. The high stability observed at high salt concentrations (80% activity at 1 M NaCl) and the activity observed even at a concentration of 4 M NaCl will allow this enzyme to function in brines typical of the food industry (Flores-Gallegos et al. 2019). Alternatively, its ability to be active in the presence of a wide variety of mono- and divalent ions, different types of detergents, and chelating ions provides the possibility of applying EstGoM in the chemical and pharmaceutical industries or for bioremediation processes (Rafeeq et al. 2022). EstGoM’s capacity to operate under extreme conditions, such as low temperature, alkaline pH, and saline environments, is closely related to the environmental parameters of its source, a 1000-m-deep water body. Therefore, EstGoM’s robust enzymatic activity could be seen as an adaptation to such environmental pressures of bacterial isolates inhabiting deep seas. In this sense, it should be noticed that some enzymes exhibit stabilization and/or activation under high pressure (HP). Although HP was not tested during the activity assays of EstGoM, it is possible that it might influence the enzymatic activity as seen in other lipases (Eisenmenger and Reyes-De-Corcuera 2009).

In conclusion, EstGoM is a new marine GDSL/SGNH AT esterase belonging to family II of lipolytic enzymes with high catalytic activity against medium-chain esters. This enzyme retains high activity over a wide range of temperatures and alkaline pH, is tolerant to high salt concentrations, and is resistant to metal ions and chemical reagents. These properties make EstGoM an attractive potential candidate for several biotechnological applications.

### Supplementary Information

Below is the link to the electronic supplementary material.

Supplementary file1 (PDF 1495 KB)

## Data Availability

No datasets were generated or analysed during the current study.

## References

[CR1] Akoh C, Lee G, Liaw Y, Huang T, Shaw J (2004). GDSL family of serine esterases/lipases. Prog Lipid Res.

[CR2] Aziz RK, Bartels D, Best AA, DeJongh M, Disz T, Edwards RA, Formsma K, Gerdes S, Glass EM, Kubal M, Meyer F, Olsen GJ, Olson R, Osterman AL, Overbeek RA, McNeil LK, Paarmann D, Paczian T, Parrello B, Pusch GD, Reich C, Stevens R, Vassieva O, Vonstein V, Wilke A, Zagnitko O (2008). The RAST server: rapid annotations using subsystems technology. BMC Genom.

[CR3] Barzkar N, Sohail M, Tamadoni Jahromi S, Gozari M, Poormozaffar S, Nahavandi R, Hafezieh M (2021). Marine bacterial esterases: emerging biocatalysts for industrial applications. Appl Biochem Biotechnol.

[CR4] Becker S, Theile S, Heppeler N, Michalczyk A, Wentzel A, Wilhelm S, Jaeger K-E, Kolmar H (2005). A generic system for the *Escherichia coli* cell-surface display of lipolytic enzymes. FEBS Lett.

[CR5] Cai X, Wang W, Lin L, He D, Huang G, Shen Y, Wei W, Wei D (2017). Autotransporter domain-dependent enzymatic analysis of a novel extremely thermostable carboxylesterase with high biodegradability towards pyrethroid pesticides. Sci Rep.

[CR6] Chandra P, Enespa SR, Arora PK (2020). Microbial lipases and their industrial applications: a comprehensive review. Microb Cell Fact.

[CR7] Cieśliński H, Białkowska AM, Długołęcka A, Daroch M, Tkaczuk KL, Kalinowska H, Kur J, Turkiewicz M (2007). A cold-adapted esterase from psychrotrophic *Pseudoalteromas* sp. strain 643A. Arch Microbiol.

[CR8] Daleke-Schermerhorn MH, Felix T, Soprova Z, Hagen-Jongman  CMT, Vikström D, Majlessi L, Beskers J, Follmann F, Punder KD, Wel NNVD, Baumgarten T, Pham TV, Piersma SR, Jiménez CR, Ulsen PV, Gier  J-WD, Leclerc C, Jong  WSP, Luirink J (2014). Decoration of outer membrane vesicles with multiple antigens by using an autotransporter approach. Appl Environ Microbiol.

[CR9] Debashish G, Malay S, Barindra S, Joydeep M, Ulber R, Le Gal Y (2005). Marine enzymes. Marine Biotechnology I.

[CR10] Eisenmenger MJ, Reyes-de-Corcuera JI (2009). High hydrostatic pressure increased stability and activity of immobilized lipase inhexane. Enzyme Microb Technol.

[CR11] Fang Z, Li J, Wang Q, Fang W, Peng H, Zhang X, Xiao Y (2014). A novel esterase from a marine metagenomic library exhibiting salt tolerance ability. J Microbiol Biotechnol.

[CR12] Flores-Gallegos AC, Delgado-García M, Ascacio-Valdés JA, Villareal-Morales S, Michel-Michel MR, Aguilar-González CN, Rodríguez-Herrera R, Kuddus M (2019). Chapter 13 - Hydrolases of halophilic origin with importance for the food industry. Enzymes in Food Biotechnology.

[CR13] Glogauer A, Martini VP, Faoro H, Couto GH, Müller-Santos M, Monteiro RA, Mitchell DA, de Souza EM, Pedrosa FO, Krieger N (2011). Identification and characterization of a new true lipase isolated through metagenomic approach. Microb Cell Fact.

[CR14] Hall TA (1999) BioEdit: a user-friendly biological sequence alignment editor and analysis program for Windows 95/98/NT. In: Nucleic acids symposium series [London]: Information Retrieval Ltd., c1979-c2000. 41:95–98

[CR15] Hausmann S, Jaeger KE, Timmis KN (2010). Lipolytic enzymes from bacteria. Handbook of hydrocarbon and lipid microbiology.

[CR16] Henderson IR, Navarro-Garcia F, Desvaux M, Fernandez RC, Ala'Aldeen D (2004). Type V protein secretion pathway: the autotransporter story. Microbiol Mol Biol Rev.

[CR17] Jong WSP, Daleke-Schermerhorn MH, Vikström D, ten Hagen-Jongman CM, de Punder K, van der Wel NN, van de Sandt CE, Rimmelzwaan GF, Follmann F, Agger EM, Andersen P, de Gier J-W, Luirink J (2014). An autotransporter display platform for the development of multivalent recombinant bacterial vector vaccines. Microb Cell Fact.

[CR18] Jose J, Chung J-W, Jeon B-J, Maas RM, Nam C-H, Pyun J-C (2009). Escherichia coli with autodisplayed Z-domain of protein A for signal amplification of SPR biosensor. Biosens Bioelectron.

[CR19] Junmei D, Tingting Y, Lianming L, Zhenrong X, Yunjuan Y, Junpei Z, Bo X, Junjun L, Zunxi H (2014). Biochemical characterization of a GDSL-Motif esterase from Bacillus sp. K91 with a new putative catalytic mechanism. J Microbiol Biotechnol.

[CR20] Kovacic F, Babic N, Krauss U, Jaeger K-E, Rojo F (2019). Classification of lipolytic enzymes from bacteria. Aerobic utilization of hydrocarbons, oils and lipids.

[CR21] Kumar S, Stecher G, Li M, Knyaz C, Tamura K (2018). MEGA X: molecular evolutionary genetics analysis across computing platforms. Mol Biol Evol.

[CR22] Leščić Ašler I, Ivić N, Kovačić F, Schell S, Knorr J, Krauss U, Wilhelm S, Kojić-Prodić B, Jaeger K-E (2010). Probing enzyme promiscuity of SGNH hydrolases. ChemBioChem.

[CR23] Meuskens I, Saragliadis A, Leo JC, Linke D (2019) Type V secretion systems: an overview of passenger domain functions. Front Microbiol 10. 10.3389/fmicb.2019.01163 10.3389/fmicb.2019.01163 PMC6555100 31214135

[CR24] Mishra SR, Panda AN, Ray L, Sahu N, Mishra G, Jadhao S, Suar M, Adhya TK, Rastogi G, Pattnaik AK, Raina V (2016). Draft genome sequence of *Pseudomonas* sp. Strain BMS12, a plant growth-promoting and protease-producing bacterium, isolated from the rhizosphere sediment of Phragmites karka of Chilika Lake. India. Genome Announc.

[CR25] Mølgaard A, Kauppinen S, Larsen S (2000). Rhamnogalacturonan acetylesterase elucidates the structure and function of a new family of hydrolases. Structure.

[CR26] Muriel-Millán LF, Millán-López S, Pardo-López L (2021). Biotechnological applications of marine bacteria in bioremediation of environments polluted with hydrocarbons and plastics. Appl Microbiol Biotechnol.

[CR27] Nicolay T, Lemoine L, Lievens E, Balzarini S, Vanderleyden J, Spaepen S (2012). Probing the applicability of autotransporter based surface display with the EstA autotransporter of *Pseudomonas stutzeri* A15. Microb Cell Fact.

[CR28] Pandurangan AP, Stahlhacke J, Oates ME, Smithers B, Gough J (2018). The SUPERFAMILY 2.0 database: a significant proteome update and a new webserver. Nucleic Acids Res.

[CR29] Petrovskaya LE, Novototskaya-Vlasova KA, Kryukova EA, Rivkina EM, Dolgikh DA, Kirpichnikov MP (2015). Cell surface display of cold-active esterase EstPc with the use of a new autotransporter from *Psychrobacter cryohalolentis* K5T. Extremophiles.

[CR30] Rafeeq H, Hussain A, Shabbir S, Ali S, Bilal M, Sher F, Iqbal HMN (2022). Esterases as emerging biocatalysts: Mechanistic insights, genomic and metagenomic, immobilization, and biotechnological applications. Biotechnol Appl Biochem.

[CR31] Rao TE, Imchen M, Kumavath R, Kim S-K, Toldrá F (2017). Chapter seven - marine enzymes: production and applications for human health. Advances in Food and Nutrition Research.

[CR32] Rodríguez-Salazar, J, Almeida-Juarez, AG, Ornelas-Ocampo, K, Millán-López, S, Raga-Carbajal, E, Rodríguez-Mejía, JL, Muriel-Millán, LF, Godoy-Lozano, EE, Rivera-Gómez, N, Rudiño-Piñera, E, and Pardo-López, L (2020) Characterization of a novel functional trimeric catechol 1,2-dioxygenase from a *Pseudomonas stutzeri* isolated from the Gulf of Mexico. Front Microbiol 11. 10.3389/fmicb.2020.01100 10.3389/fmicb.2020.01100 PMC7287156 32582076

[CR33] Rojas-Vargas J, Muriel-Millán, and Pardo-López, L,  (2023). Draft genome sequence of *Pseudomonas* sp. GOM6, a lipolytic strain isolated from seawater of the Gulf of Mexico. Microbiol Resourc Announc.

[CR34] Sana B, Kim S-K (2015). Marine microbial enzymes: current status and future prospects. Springer Handbook of Marine Biotechnology.

[CR35] Schneider CA, Rasband WS, Eliceiri KW (2012). NIH Image to ImageJ: 25 years of image analysis. Nat Methods.

[CR36] Shakiba MH, Ali MSM, Rahman RNZRA, Salleh AB, Leow TC (2016). Cloning, expression and characterization of a novel cold-adapted GDSL family esterase from *Photobacterium* sp. strain J15. Extremophiles.

[CR37] St. Laurent JB, de Buzzaccarini F, De Clerck K, Demeyere H, Labeque R, Lodewick R, van Langenhove L (2007) B.1.I - Laundry cleaning of textiles. In: Johansson I, Somasundaran P (eds) Handbook for Cleaning/Decontamination of Surfaces. Elsevier Science B.V., Amsterdam, pp 57–102

[CR38] Talker-Huiber D, Jose J, Glieder A, Pressnig M, Stubenrauch G, Schwab H (2003). Esterase EstE from *Xanthomonas vesicatoria* (Xv_EstE) is an outer membrane protein capable of hydrolyzing long-chain polar esters. Appl Microbiol Biotechnol.

[CR39] Tozakidis IEP, Lüken LM, Üffing A, Meyers A, Jose J (2020). Improving the autotransporter-based surface display of enzymes in *Pseudomonas putida* KT2440. Microb Biotechnol.

[CR40] Trincone A (2010). Potential biocatalysts originating from sea environments. J Mol Catal B Enzym.

[CR41] van Ulsen P, Zinner KM, Jong WSP, Luirink J (2018) On display: autotransporter secretion and application. FEMS Microbiol Lett 365(18). 10.1093/femsle/fny165 10.1093/femsle/fny165 30085010

[CR42] van den Berg B (2010). Crystal structure of a full-length autotransporter. J Mol Biol.

[CR43] Waterhouse A, Bertoni M, Bienert S, Studer G, Tauriello G, Gumienny R, Heer FT, de Beer TAP, Rempfer C, Bordoli L, Lepore R, Schwede T (2018). SWISS-MODEL: homology modelling of protein structures and complexes. Nucleic Acids Res.

[CR44] Wicka M, Wanarska M, Krajewska E, Pawlak-Szukalska A, Kur J, Cieśliński H (2016). Cloning, expression, and biochemical characterization of a cold-active GDSL-esterase of a *Pseudomonas* sp. S9 isolated from Spitsbergen island soil. Acta Biochim Pol.

[CR45] Wilhelm S, Tommassen J, Jaeger K-E (1999). A novel lipolytic enzyme located in the outer membrane of *Pseudomonas aeruginosa*. J Bacteriol.

[CR46] Wilhelm S, Gdynia A, Tielen P, Rosenau F, Jaeger K-E (2007). The autotransporter esterase EstA of *Pseudomonas aeruginosa* is required for rhamnolipid production, cell motility, and biofilm formation. J Bacteriol.

[CR47] Wilhelm S, Rosenau F, Becker S, Buest S, Hausmann S, Kolmar H, Jaeger K-E (2007). Functional cell-surface display of a lipase-specific chaperone. ChemBioChem.

[CR48] Wilhelm S, Rosenau F, Kolmar H, Jaeger K-E (2011). Autotransporters with GDSL passenger domains: molecular physiology and biotechnological applications. ChemBioChem.

[CR49] Wu G, Wu G, Zhan T, Shao Z, Liu Z (2013). Characterization of a cold-adapted and salt-tolerant esterase from a psychrotrophic bacterium Psychrobacterpacificensis. Extremophiles.

[CR50] Yang TH, Kwon M-A, Song JK, Pan JG, Rhee JS (2010). Functional display of *Pseudomonas* and *Burkholderia* lipases using a translocator domain of EstA autotransporter on the cell surface of *Escherichia coli*. J Biotechnol.

[CR51] Yang Z, Zhang Y, Shen T, Xie Y, Mao Y, Ji C (2013). Cloning, expression and biochemical characterization of a novel, moderately thermostable GDSL family esterase from *Geobacillus thermodenitrificans* T2. J Biosci Bioeng.

[CR52] Zhang C, Kim S-K (2010). Research and application of marine microbial enzymes: status and prospects. Mar Drugs.

