# Supplementary material for: A Novel Thermo-Alkaline Stable GDSL/SGNH Esterase with Broad Substrate Specificity from a Deep-Sea Pseudomonas sp
Source: Mar Biotechnol (NY). 2024 May 1;26(3):447–59. doi: 10.1007/s10126-024-10308-w (PMC11178605; doi:10.1007/s10126-024-10308-w)
Supplement: Supplementary file 1 — Supplementary file1 (PDF 1495 KB) [file 10126_2024_10308_MOESM1_ESM.pdf]

Supplementary information

A novel thermo-alkaline stable GDSL/SGNH esterase with broad substrate specificity from a deep-sea *Pseudomonas* sp.

José Luis Rodríguez-Mejía[1a†], Itzel Anahí Hidalgo-Manzano[1†], Luis Felipe Muriel-Millán[1†], Nancy Rivera-Gomez[1b], Diana X. Sahonero-Canavesi[1c], Edmundo Castillo[2] and Liliana Pardo-López[1]

[1]Departamento de Microbiología Molecular, Instituto de Biotecnología, Universidad Nacional Autónoma de México, Av. Universidad 2001, Col. Chamilpa, Cuernavaca, Morelos 62210, México

[2]Departamento de Ingeniería Celular y Biocatálisis, Instituto de Biotecnología, Universidad Nacional Autónoma de México, Av. Universidad 2001, Col. Chamilpa, Cuernavaca, Morelos 62210, México

[a]Present address: Edificio Dr. Carlos Méndez, Centro Universitario de Investigaciones Biomédicas, Universidad de Colima, Campus Central Colima; Avenida 25 de julio #965, col. V. Sn. Sebastián, C.P. 28045, Colima, Colima, México

[b]Present address: IPN: CICATA Unidad Morelos del Instituto Politécnico Nacional. Blvd. de la Tecnologia 1036-P 2/2, 62790 Atlacholoaya, Morelos, México

[c]Present address: NIOZ Royal Netherlands Institute for Sea Research, Department of Marine Microbiology and Biogeochemistry, P.O. Box 59, 1797AB Den Burg, Texel, The Netherlands.

[†]These authors have contributed equally to this work

Corresponding author:

e-mail: liliana.pardo@ibt.unam.mx, edmundo.castillo@ibt.unam.mx

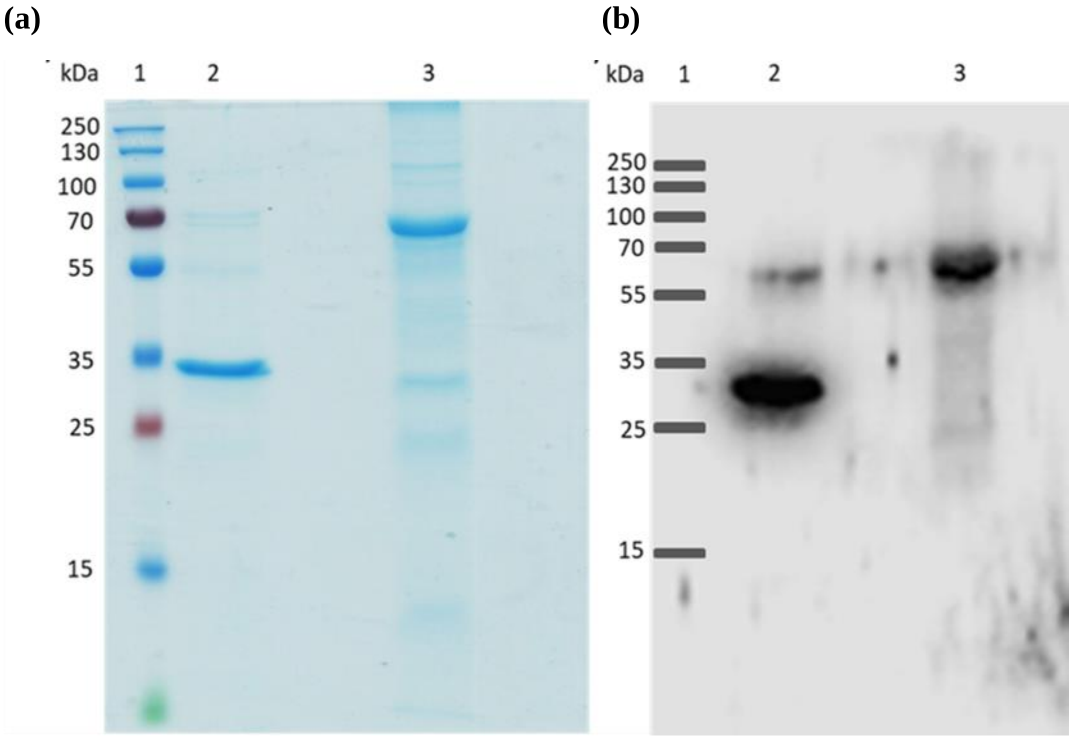

Figure S1. Purification of EstGoM enzyme. **(a)** SDS–PAGE of purified 6xHis-tagged EstGoM (67 kDa) (lane 3). Lane 1; Protein molecular weight marker (Thermo Scientific™). Lane 2; A purified 6xHis-tagged catechol 1,2 dioxygenase (Rodríguez-Salazar *et al.*, 2020) was used as positive control for western Blot detection of EstGoM. **(b)** Confirmation of the purification of EstGoM by western blot assays. An anti-6xHis tag antibody (Abcam, UK) was used at 1:10000 dilution. The lanes are the same as those from panel A.

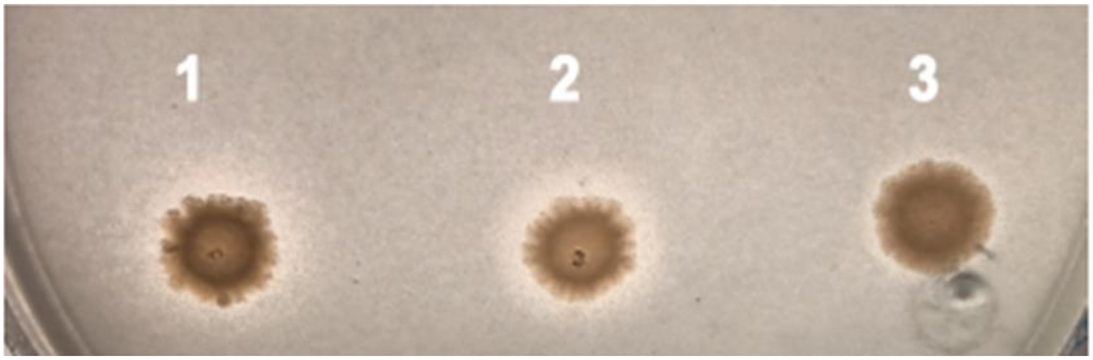

Figure S2. *E. coli* colonies surrounded by clear halos compatible with lipolytic activity. The clones were grown on modified LB agar plates with 1% tributyrin and incubated for 48 h at 30°C. Clone 3 harbors the EstGoM coding gene.

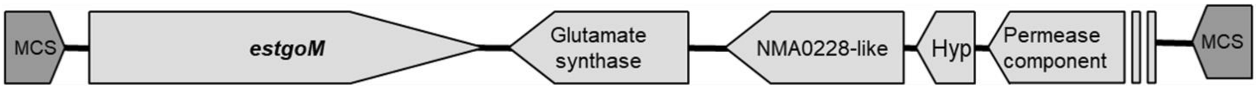

Figure S3. Schematic representation of the GOM6 genomic region cloned into the pUC19 vector obtained from *E. coli* clone 3 shown in Figure A2. MCS; Multiple cloning site of pUC19 vector.

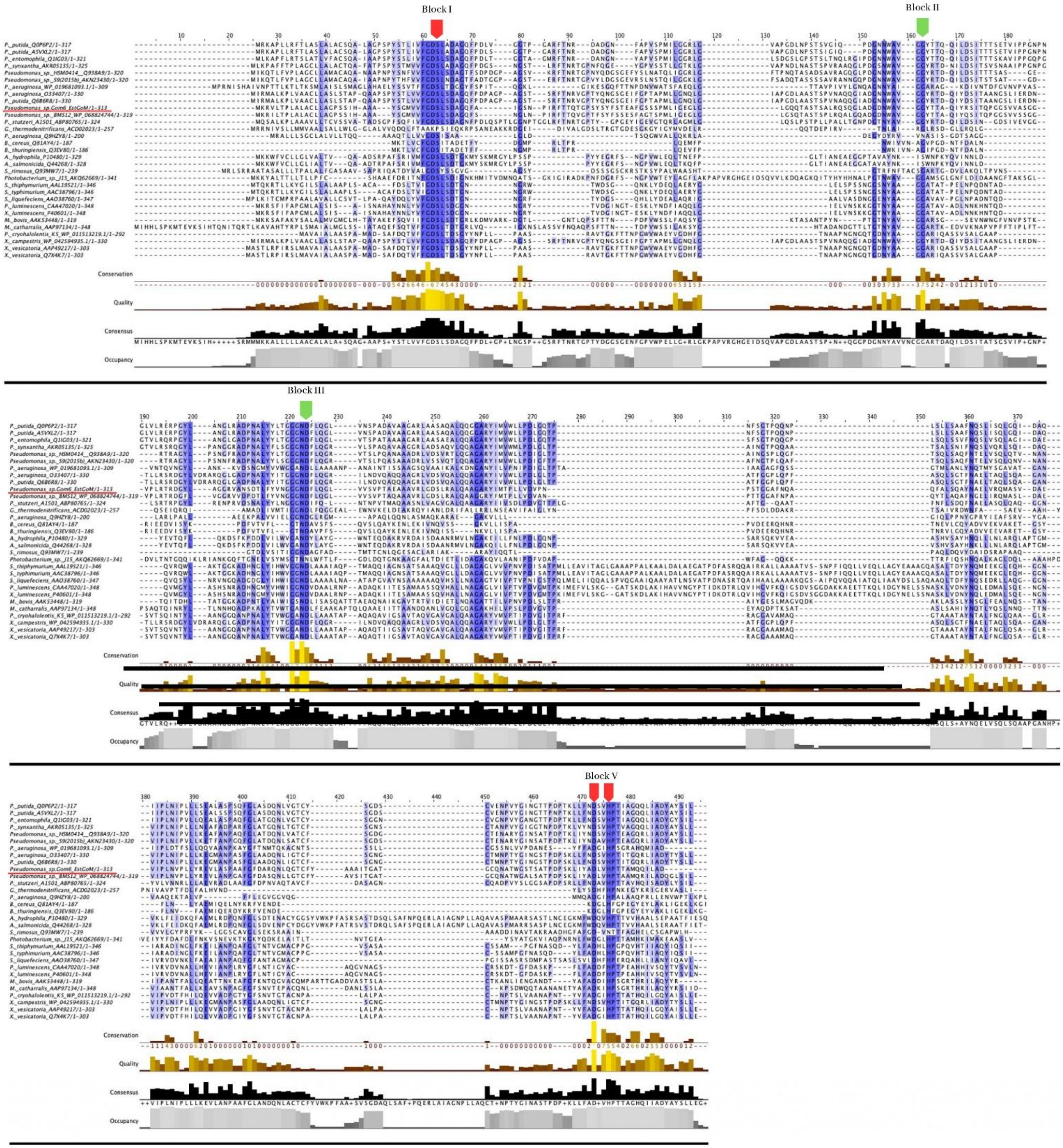

Figure S4. Amino acid sequence alignment of EstGoM (redline) and homologous esterases. The alignment was made using only the amino acids of the esterase domain and signal peptide. It shows the four blocks that conserve the catalytic amino acids; blocks I, II, III, and V. Red arrowheads indicate the amino acids of the catalytic triad (S, D, H). Green arrowheads designate the amino acids G, and N that act as hydrogen-bond donors to the oxyanion hole (Mølgaard *et al.*, 2000). The Jalview program version 2.11.1.3 was used to visualize the multiple alignments.

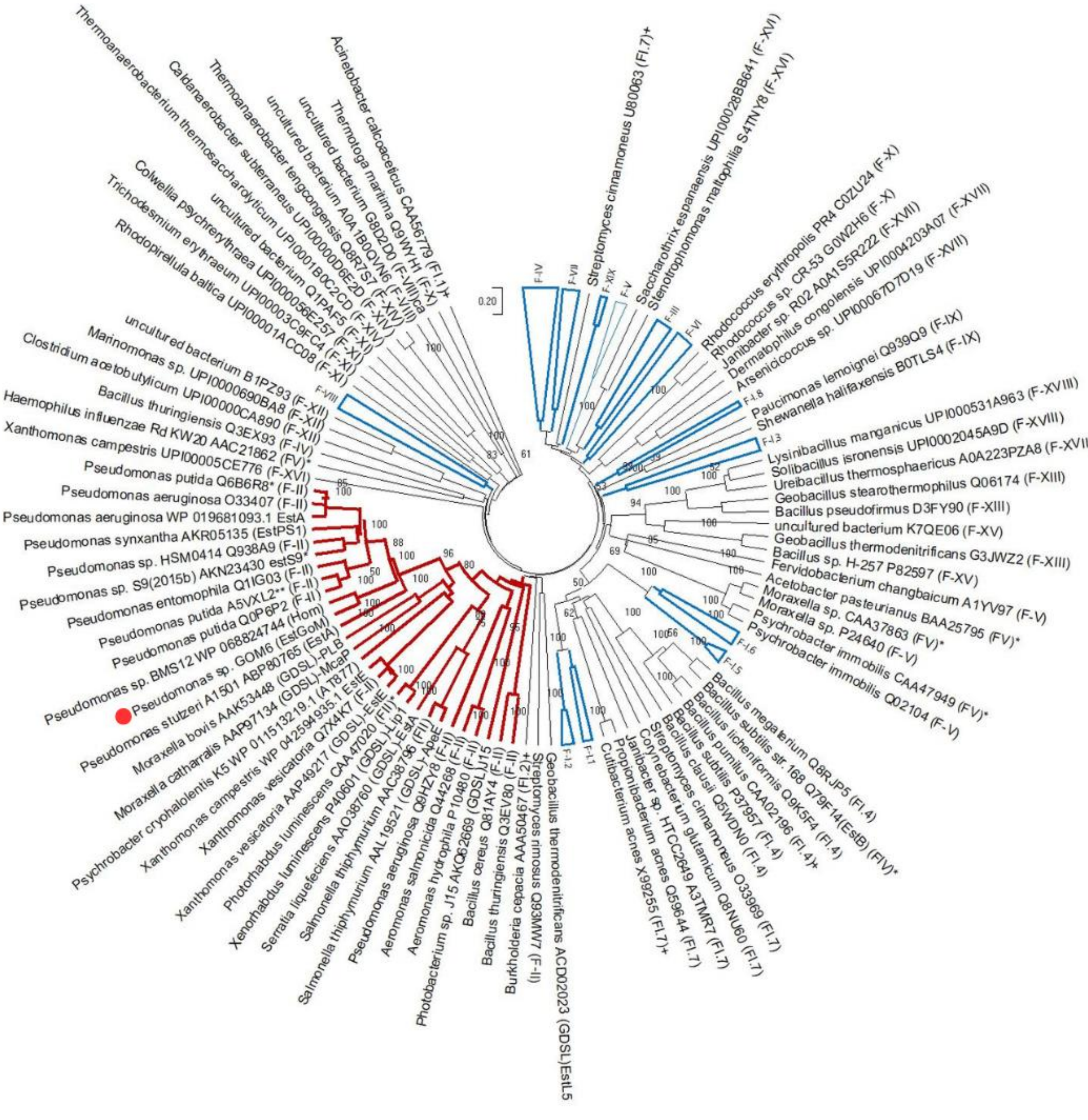

Figure S5. Phylogenetic tree of the amino acid sequences of EstGoM and other lipolytic enzymes from all 19 presently described families. The sequences were analyzed by the UPGMA method using the MEGA X software. The red circle denotes the amino acid sequence of EstGoM, which belongs to family II of lipolytic enzymes (branches in red).

(a) **(b)**

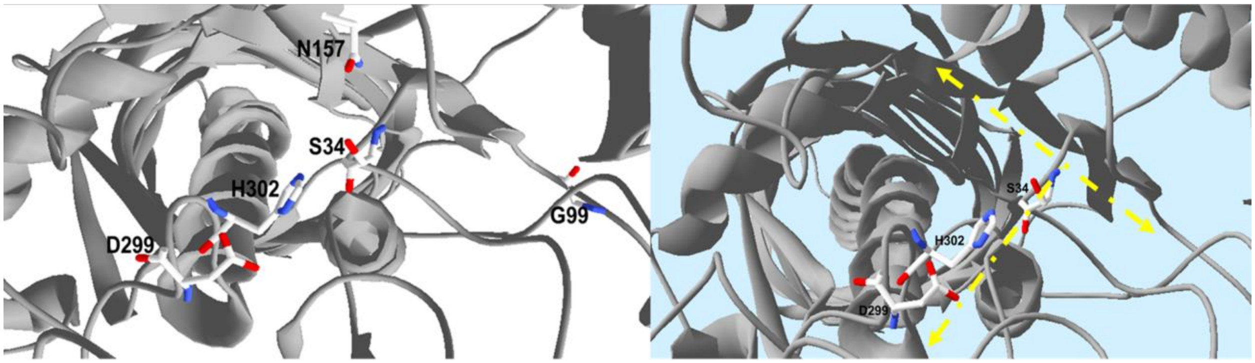

Figure S6. 3D image obtained using DeepView/Swiss-PdbViewer by Swiss Institute of Bioinformatics v4.1.0. It´s show a close view of the catalytic site of EstGoM where the catalytic triad formed by the amino acids Ser34, His302 and Asp299. **(a)** The image illustrates the catalytic triad plus two amino acids more G99 and N157 that contributing as extra proton donor into de oxyanion conformation, a typical array by the SGNH members. **(b)** The catalytic triad has a perpendicular orientation as is shown by the yellow arrows, a particular of the SGNH hydrolases family

Table S1. Homologous proteins of EstGoM identified by BLASTP search. The results were obtained in a search performed at https://blast.ncbi.nlm.nih.gov/ on February 21st 2023

Description	Scientific Name	Max Score	Total Score	Query Cover	E value	Per. Ident	Accesion Length	Accession
autotransporter domain-containing SGNH/GDSL hydrolase family protein [Pseudomonas sp. BMS12]	Pseudomonas sp. BMS12	1078	1078	100%	0.0	84.04%	633	WP_068824744.1
autotransporter domain-containing SGNH/GDSL hydrolase family protein [Pseudomonas sp. PDM14]	Pseudomonas sp. PDM14	933	933	100%	0.0	73.82%	631	WP_192328832.1
autotransporter domain-containing SGNH/GDSL hydrolase family protein [Pseudomonas insulae]	Pseudomonas insulae	880	880	96%	0.0	74.59%	634	WP_205348293.1
autotransporter domain-containing SGNH/GDSL hydrolase family protein [Pseudomonas mangrovi]	Pseudomonas mangrovi	854	854	100%	0.0	67.24%	636	WP_199286993.1
autotransporter domain-containing esterase [Pseudomonas mangrovi]	Pseudomonas mangrovi	854	854	100%	0.0	67.24%	635	PTU75081.1
autotransporter domain-containing SGNH/GDSL hydrolase family protein [Pseudomonas sp. PDM15]	Pseudomonas sp. PDM15	789	789	100%	0.0	65.14%	644	WP_192399213.1
autotransporter domain-containing SGNH/GDSL hydrolase family protein [Pseudomonas sp. YIM B01952]	Pseudomonas sp. YIM B01952	781	781	100%	0.0	64.01%	642	WP_252271801.1
autotransporter domain-containing protein [Pseudomonas sp. PDM16]	Pseudomonas sp. PDM16	767	767	100%	0.0	63.19%	646	MBD9413119.1
autotransporter domain-containing SGNH/GDSL hydrolase family protein [Pseudomonas sp. PDM16]	Pseudomonas sp. PDM16	767	767	100%	0.0	63.19%	645	WP_225580199.1
autotransporter domain-containing SGNH/GDSL hydrolase family protein [Pseudomonas sp. F(2018)]	Pseudomonas sp. F(2018)	766	766	100%	0.0	64.26%	646	WP_137973569.1
autotransporter domain-containing SGNH/GDSL hydrolase family protein [Pseudomonas paralcaligenes]	Pseudomonas paralcaligenes	741	741	100%	0.0	62.37%	645	WP_220812707.1

autotransporter domain-containing SGNH/GDSL hydrolase family protein [Pseudomonas ullengensis]	Pseudomonas ullengensis	739	739	100%	0.0	61.16%	644	WP_183090321.1
autotransporter domain-containing protein [Pseudomonas sp. Gutcm_11s]	Pseudomonas sp. Gutcm_11s	736	736	100%	0.0	62.79%	643	WP_273815894.1
autotransporter domain-containing SGNH/GDSL hydrolase family protein [Pseudomonas sp. ML96]	Pseudomonas sp. ML96	734	734	100%	0.0	61.41%	643	WP_043311040.1
hypothetical protein [Pseudomonas alcaligenes]	Pseudomonas alcaligenes	699	699	97%	0.0	59.25%	645	MBC9252505.1
autotransporter domain-containing SGNH/GDSL hydrolase family protein [Pseudomonas alcaligenes]	Pseudomonas alcaligenes	699	699	97%	0.0	59.25%	643	WP_262410624.1
autotransporter domain-containing SGNH/GDSL hydrolase family protein [Pseudomonas]	Pseudomonas	645	645	100%	0.0	54.26%	633	WP_069898416.1
autotransporter domain-containing SGNH/GDSL hydrolase family protein [Pseudomonas sp. 5P_3.1_Bac2]	Pseudomonas sp. 5P_3.1_Bac2	644	644	100%	0.0	54.22%	634	WP_262182708.1
autotransporter domain-containing SGNH/GDSL hydrolase family protein [Pseudomonas marincola]	Pseudomonas marincola	643	643	100%	0.0	54.11%	633	WP_197737962.1
outer membrane lipase/esterase [Pseudomonas segetis]	Pseudomonas segetis	639	639	100%	0.0	52.56%	633	SNS35020.1
autotransporter domain-containing SGNH/GDSL hydrolase family protein [Pseudomonas marincola]	Pseudomonas marincola	638	638	100%	0.0	53.95%	633	WP_212633016.1
autotransporter domain-containing SGNH/GDSL hydrolase family protein [Pseudomonas sp. MS19]	Pseudomonas sp. MS19	638	638	100%	0.0	53.64%	633	WP_201970334.1
autotransporter domain-containing SGNH/GDSL hydrolase family protein [Pseudomonas carbonaria]	Pseudomonas carbonaria	632	632	100%	0.0	53.55%	635	WP_187670663.1
autotransporter domain-containing esterase [Pseudomonadaceae bacterium]	Pseudomonadaceae bacterium	620	620	98%	0.0	53.87%	621	MAB98977.1
autotransporter domain-containing esterase [Pseudomonadaceae bacterium]	Pseudomonadaceae bacterium	618	618	98%	0.0	53.40%	621	MBQ56696.1

autotransporter domain-containing protein [Pseudomonadaceae bacterium]	Pseudomonadaceae bacterium	618	618	100%	0.0	52.25%	632	MBX9713638.1
autotransporter domain-containing esterase [Pseudomonas sp. S9]	Pseudomonas sp. S9	617	617	98%	0.0	51.82%	621	WP_010488574.1
autotransporter domain-containing SGNH/GDSL hydrolase family protein [Pseudomonas sp. M30-35]	Pseudomonas sp. M30-35	615	615	98%	0.0	51.97%	621	WP_087519207.1
autotransporter domain-containing SGNH/GDSL hydrolase family protein [Pseudomonas mangiferae]	Pseudomonas mangiferae	615	615	96%	0.0	53.53%	633	WP_143488498.1
autotransporter domain-containing SGNH/GDSL hydrolase family protein [Pseudomonas segetis]	Pseudomonas segetis	615	615	98%	0.0	51.82%	621	WP_089359883.1
TPA: autotransporter domain-containing esterase [Pseudomonas sp.]	Pseudomonas sp.	614	614	100%	0.0	54.88%	633	HBX54383.1
autotransporter domain-containing protein [Pseudomonas sp. MS19]	Pseudomonas sp. MS19	612	612	98%	0.0	52.92%	621	NRH27794.1
autotransporter domain-containing SGNH/GDSL hydrolase family protein [Pseudomonas sp. LS44]	Pseudomonas sp. LS44	611	611	100%	0.0	51.31%	635	WP_258252814.1
autotransporter domain-containing protein [Pseudomonadales bacterium]	Pseudomonadales bacterium	610	610	100%	0.0	50.83%	651	MBH2034619.1
autotransporter domain-containing protein [Pseudomonas panipatensis]	Pseudomonas panipatensis	609	609	100%	0.0	51.00%	643	WP_090265317.1
autotransporter domain-containing protein [Pseudomonadaceae bacterium]	Pseudomonadaceae bacterium	608	608	100%	0.0	51.17%	632	MBX9754959.1
autotransporter domain-containing protein [Pseudomonas indica]	Pseudomonas indica	603	603	100%	0.0	53.11%	650	MBU3055085.1
autotransporter domain-containing esterase [Pseudomonas thermotolerans]	Pseudomonas thermotolerans	602	602	96%	0.0	52.74%	637	WP_036987269.1
autotransporter domain-containing esterase [Gammaproteobacteria bacterium]	Gammaproteobacteria bacterium	602	602	96%	0.0	52.73%	633	MBO2509121.1

autotransporter domain-containing esterase [Pseudomonas thermotolerans]	Pseudomonas thermotolerans	602	602	96%	0.0	52.74%	637	WP_040640609.1
autotransporter domain-containing SGNH/GDSL hydrolase family protein [Pseudomonas alcaligenes]	Pseudomonas alcaligenes	601	601	96%	0.0	52.57%	634	WP_263147060.1
autotransporter domain-containing SGNH/GDSL hydrolase family protein [Pseudomonas indica]	Pseudomonas indica	600	600	100%	0.0	52.66%	651	WP_095649336.1
autotransporter domain-containing SGNH/GDSL hydrolase family protein [Pseudomonas indica]	Pseudomonas indica	599	599	100%	0.0	52.66%	651	WP_084333013.1
autotransporter domain-containing SGNH/GDSL hydrolase family protein [Pseudomonas benzenivorans]	Pseudomonas benzenivorans	592	592	100%	0.0	51.90%	651	WP_245729624.1
autotransporter domain-containing SGNH/GDSL hydrolase family protein [Pseudomonas psychrotolerans]	Pseudomonas psychrotolerans	590	590	96%	0.0	52.33%	645	WP_058761477.1
esterase EstA [Pseudomonas aeruginosa]	Pseudomonas aeruginosa	589	589	100%	0.0	49.77%	646	WP_116827783.1
TPA: esterase EstA [Pseudomonas aeruginosa]	Pseudomonas aeruginosa	589	589	100%	0.0	49.62%	646	HCF6607617.1
esterase EstA [Pseudomonas aeruginosa]	Pseudomonas aeruginosa	588	588	100%	0.0	49.77%	646	WP_124125165.1
autotransporter domain-containing SGNH/GDSL hydrolase family protein [Pseudomonas alcaligenes]	Pseudomonas alcaligenes	588	588	99%	0.0	50.54%	639	WP_110683411.1
esterase EstA [Pseudomonas aeruginosa]	Pseudomonas aeruginosa	588	588	100%	0.0	49.62%	646	WP_128528463.1
TPA: esterase EstA [Pseudomonas aeruginosa]	Pseudomonas aeruginosa	588	588	100%	0.0	49.62%	646	HCE8943354.1
esterase EstA [Pseudomonas aeruginosa]	Pseudomonas aeruginosa	588	588	100%	0.0	49.62%	646	WP_125878398.1

esterase EstA [Pseudomonas aeruginosa]	Pseudomonas aeruginosa	588	588	100%	0.0	49.46%	646	WP_263957143.1
esterase EstA [Pseudomonas aeruginosa]	Pseudomonas aeruginosa	587	587	100%	0.0	49.62%	646	MBG5299325.1
esterase EstA [Pseudomonas aeruginosa]	Pseudomonas aeruginosa	587	587	100%	0.0	49.62%	646	MBG4728849.1
esterase EstA [Pseudomonas aeruginosa]	Pseudomonas aeruginosa	587	587	100%	0.0	49.62%	646	WP_121328293.1
esterase EstA [Pseudomonas aeruginosa]	Pseudomonas aeruginosa	587	587	100%	0.0	49.62%	646	WP_058173405.1
esterase EstA [Pseudomonas aeruginosa]	Pseudomonas aeruginosa	587	587	100%	0.0	49.46%	646	WP_124199860.1
esterase EstA [Pseudomonas aeruginosa]	Pseudomonas aeruginosa	587	587	100%	0.0	49.62%	646	MBH9419440.1
esterase EstA [Pseudomonas aeruginosa]	Pseudomonas aeruginosa	587	587	100%	0.0	49.62%	646	WP_116844085.1
autotransporter domain-containing protein [Pseudomonas aeruginosa]	Pseudomonas aeruginosa	587	587	100%	0.0	49.62%	646	MCO1740317.1
esterase EstA [Pseudomonas aeruginosa]	Pseudomonas aeruginosa	587	587	100%	0.0	49.62%	646	WP_121395021.1
esterase EstA [Pseudomonas aeruginosa]	Pseudomonas aeruginosa	587	587	100%	0.0	49.62%	646	MBG7304461.1
esterase EstA [Pseudomonas aeruginosa]	Pseudomonas aeruginosa	587	587	100%	0.0	49.46%	646	WP_033956335.1
esterase EstA [Pseudomonas aeruginosa]	Pseudomonas aeruginosa	587	587	100%	0.0	49.62%	646	WP_057386155.1
esterase EstA [Pseudomonas aeruginosa]	Pseudomonas aeruginosa	587	587	100%	0.0	49.46%	646	MBG3934767.1

esterase EstA [Pseudomonas aeruginosa]	Pseudomonas aeruginosa	587	587	100%	0.0	49.62%	646	WP_116801582.1
esterase EstA [Pseudomonas aeruginosa]	Pseudomonas aeruginosa	587	587	100%	0.0	49.46%	646	WP_269972437.1
esterase EstA [Pseudomonas aeruginosa]	Pseudomonas aeruginosa	587	587	100%	0.0	49.62%	646	WP_132911996.1
TPA: autotransporter domain-containing protein [Pseudomonas aeruginosa]	Pseudomonas aeruginosa	587	587	100%	0.0	49.62%	646	HBP6405861.1
TPA: esterase EstA [Pseudomonas aeruginosa]	Pseudomonas aeruginosa	587	587	100%	0.0	49.62%	646	HCA5813645.1
autotransporter domain-containing esterase [Pseudomonas sp. TTU2014-080ASC]	Pseudomonas sp. TTU2014-080ASC	587	587	100%	0.0	51.47%	633	WP_058069088.1
esterase EstA [Pseudomonas aeruginosa]	Pseudomonas aeruginosa	587	587	100%	0.0	49.46%	646	WP_057390052.1
esterase EstA [Pseudomonas aeruginosa]	Pseudomonas aeruginosa	587	587	100%	0.0	49.31%	646	WP_124126631.1
esterase EstA [Pseudomonas aeruginosa]	Pseudomonas aeruginosa	587	587	100%	0.0	49.46%	646	MBG4753524.1
esterase EstA [Pseudomonas aeruginosa]	Pseudomonas aeruginosa	587	587	100%	0.0	49.46%	646	WP_113887050.1
esterase EstA [Pseudomonas aeruginosa]	Pseudomonas aeruginosa	587	587	100%	0.0	49.46%	646	WP_112207983.1
esterase EstA [Pseudomonas aeruginosa]	Pseudomonas aeruginosa	587	587	100%	0.0	49.46%	646	MBG7458728.1
esterase EstA [Pseudomonas aeruginosa]	Pseudomonas aeruginosa	587	587	100%	0.0	49.62%	646	WP_003101796.1
TPA: esterase EstA [Pseudomonas aeruginosa]	Pseudomonas aeruginosa	587	587	100%	0.0	49.62%	646	HCE9279091.1

esterase EstA [Pseudomonas aeruginosa]	Pseudomonas aeruginosa	587	587	100%	0.0	49.62%	646	EJO5053589.1
esterase EstA [Pseudomonas aeruginosa]	Pseudomonas aeruginosa	587	587	100%	0.0	49.62%	646	WP_023119880.1
TPA: esterase EstA [Pseudomonas aeruginosa]	Pseudomonas aeruginosa	587	587	100%	0.0	49.46%	646	HCF1643813.1
esterase EstA [Pseudomonas aeruginosa]	Pseudomonas aeruginosa	587	587	100%	0.0	49.62%	646	WP_043083073.1
esterase EstA [Pseudomonas aeruginosa]	Pseudomonas aeruginosa	587	587	100%	0.0	49.46%	646	WP_121572985.1
esterase EstA [Pseudomonas aeruginosa]	Pseudomonas aeruginosa	587	587	100%	0.0	49.46%	646	MBG6473784.1
esterase EstA [Pseudomonas aeruginosa]	Pseudomonas aeruginosa	586	586	100%	0.0	49.46%	646	WP_033995332.1
esterase EstA [Pseudomonas aeruginosa]	Pseudomonas aeruginosa	586	586	100%	0.0	49.46%	646	WP_033943227.1
esterase EstA [Pseudomonas aeruginosa]	Pseudomonas aeruginosa	586	586	100%	0.0	49.38%	646	WP_073667237.1
TPA: esterase EstA [Pseudomonas aeruginosa]	Pseudomonas aeruginosa	586	586	100%	0.0	49.62%	646	HBO5338726.1
esterase EstA [Pseudomonas aeruginosa]	Pseudomonas aeruginosa	586	586	100%	0.0	49.46%	646	WP_103758221.1
esterase EstA [Pseudomonas aeruginosa]	Pseudomonas aeruginosa	586	586	100%	0.0	49.46%	646	WP_257367294.1
esterase EstA [Pseudomonas aeruginosa]	Pseudomonas aeruginosa	586	586	100%	0.0	49.62%	646	WP_116802991.1
TPA: esterase EstA [Pseudomonas aeruginosa]	Pseudomonas aeruginosa	586	586	100%	0.0	49.46%	646	HBO4502108.1

esterase EstA [Pseudomonas aeruginosa]	Pseudomonas aeruginosa	586	586	100%	0.0	49.46%	646	WP_116851236.1
esterase EstA [Pseudomonas aeruginosa]	Pseudomonas aeruginosa	586	586	100%	0.0	49.46%	646	WP_096069185.1
esterase EstA [Pseudomonas aeruginosa]	Pseudomonas aeruginosa	586	586	100%	0.0	49.46%	646	WP_125883238.1
esterase EstA [Pseudomonas aeruginosa]	Pseudomonas aeruginosa	586	586	100%	0.0	49.46%	646	WP_093944949.1
autotransporter domain-containing protein [Pseudomonas aeruginosa]	Pseudomonas aeruginosa	586	586	100%	0.0	49.46%	646	MCO3547721.1
esterase EstA [Pseudomonas aeruginosa]	Pseudomonas aeruginosa	586	586	100%	0.0	49.46%	646	WP_058164439.1

Table S2. Purification of EstGoM

Purification steps	Volume (mL)	Volumetric activity (U/mL)	Total protein (mg/mL)	Specific activity (U/mg)	Total activity (U)	Purification factor	Yield (%)
Crude extract	6.0	1.23	5.6	0.22	7.35	1	100
Purified protein	0.4	2.67	0.5	5.35	1.07	24	15
